# Utilization of H-bond interaction of nucleobase Uralic with antitumor methotrexate to design drug carrier with ultrahigh loading efficiency and pH-responsive drug release

**DOI:** 10.1093/rb/rbu010

**Published:** 2014-10-20

**Authors:** Teng-Teng Cai, Qi Lei, Bin Yang, Hui-Zhen Jia, Hong Cheng, Li-Han Liu, Xuan Zeng, Jun Feng, Ren-Xi Zhuo, Xian-Zheng Zhang

**Affiliations:** Key Laboratory of Biomedical Polymers of Ministry of Education and Department of Chemistry, Wuhan University, Wuhan 430072, China

**Keywords:** nucleobase Uralic, H-bond interaction, linear-hyperbranched copolymer, drug loading efficiency, pH-sensitivity

## Abstract

A novel Uralic (U)-rich linear-hyperbranched mono-methoxy poly (ethylene glycol)-hyperbranched polyglycerol-graft-Uralic (mPEG-HPG-g-U) nanoparticle (NP) was prepared as drug carrier for antitumor methotrexate (MTX). Due to the H-bond interaction of U with MTX and hydrophobic interaction, this NP exhibited high drug loading efficiency of up to 40%, which was significantly higher than that of traditional NPs based on U-absent copolymers (<15%). In addition, MTX-loaded mPEG-HPG-g-U NPs also demonstrated an acidity-accelerated drug release behavior.

## Introduction

The last decade has witnessed the rapid development of nanoparticle (NP)-aided drug delivery [1–4]. NPs, represented by micellar NPs (mNPs), have proven effective to improve the solubility, pharmacokinetics, efficacy and biosafety of poorly water-soluble drugs. In particular, NPs exhibit an outstanding advantage for tumor chemotherapy that they can mediate passive accumulation of loaded drugs at tumor tissues [5–7]. In most cases, loading drug into mNPs relies on the hydrophobic interaction of drug molecules with the core-forming components of mNPs, which has become the preferred strategy for drug delivery during the last decade. Nevertheless, the loading efficiencies are far from satisfactory, mostly less than 10% [8–11]. When used *in vivo*, a high dose of drug-loaded NPs is therefore needed to enable effective chemotherapy, while leading to the risk of thrombosis/embolism caused by the inter-particle aggregation [12].

Specific design of NPs according to the intrinsic characteristics of a drug agent represents the future trend of developing drug delivery systems, especially for the first-line drugs [11, 13]. This strategy may offer a marked property improvement beyond expectation. For instance, Messersmith group reported a polymeric pro-drug of bortezomib clinically used for multiple myeloma treatment, in which boronic acid-containing bortezomib can spontaneously attach to a catechol-grafted polymer via covalent boronate linking for lysosomal acidity-targeting pH-responsive drug release [13]. Hedrick *et al.* [11] described the fabrication of a urea-rich mNP with the substantially enhanced loading capacity of antitumor doxorubicin (DOX) by taking advantage of hydrogen bonds formed between DOX and urea groups.

Methotrexate (MTX) is a frequently used antitumor agent approved for the treatment of many cancers including breast, skin, head and neck, as well as lung [14–16]. However, there remain some problems to be solved for the clinical application. MTX cannot but be taken no more than twice per week owing to the serious, even life-threatening side effects [17]. In addition, the intravenous administration of MTX is limited by its poor water solubility. Therefore, it is of significant importance to establish an efficient MTX delivery nanosystem.

**Scheme 1.**
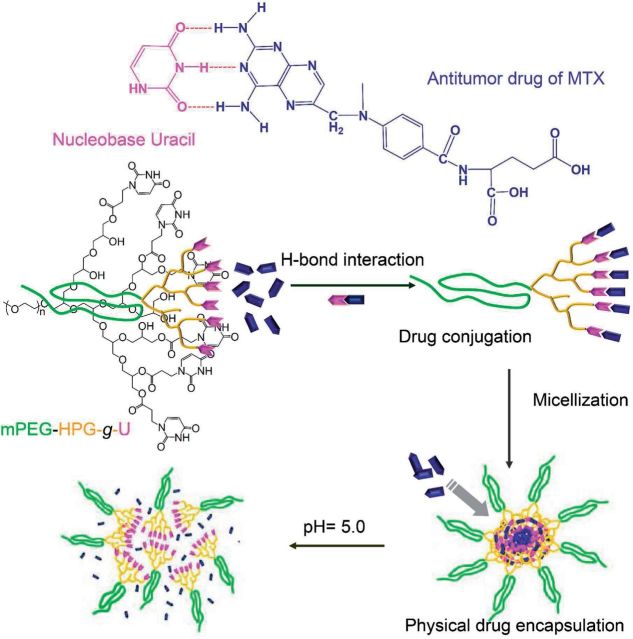
schematic illustration of mPEG-HPG-*g*-U for enhanced MTX-loading efficiency and pH-responsive MTX release by virtue of H-bond interaction of MTX with Uralic.

As known, DNA strands are linked together in parallel by specific base-pairing mechanism [18, 19]. The involved multiple hydrogen bonds play a predominant role in maintaining the structural stability. Recently, nucleobase is identified to be able to non-covalently bind with 2,6-diaminopyridine (DAP) through complementary multiple H-bonds [20–22]. Inspired by these findings, this study described a delicate design of a polymeric nanovehicle for the achievement of highly enhanced MTX-loading capacity, by virtue of the ability of DAP-bearing MTX to form H-bonds with nucleobase Uralic (Scheme 1). Herein, we chemically incorporated nucleobase Uralic onto the terminal of monomethoxy poly (ethylene glycol)-hyperbranched polyglycerol (mPEG-HPG), affording U-rich linear-hyperbranched copolymer of mPEG-HPG-g-U. Owing to the clustering effect, the enrichment of Uralic functionality would facilitate the active capture of MTX molecules onto polymeric matrix through H-bond interaction. Meanwhile, the enhanced hydrophobicity/hydrophilicity balance ratio after U conjugation would induce the self-aggregation of mPEG-HPG-*g*-U/MTX complexes into mNPs, which is able to further physically encapsulate MTX through hydrophobic interaction. The hyperbranched architecture is favorable for the encapsulation pathway due to the well-known ‘dendritic box’ effect [23]. The integration of two loading mechanisms into one delivery system is expected to offer high MTX-loading capacity. Interestingly, MTX-loaded mNPs were further revealed to present an acidity-accelerated release behavior possibly due to the acidity-induced destruction of hydrogen bonds. This finding sounds appealing since it would benefit tumor chemotherapy in consideration of the substantial pH decline in tumor tissue/cells when compared with physiologically normal conditions [24, 25].

## Experimental Section

### Materials

Dimethylsulfoxide (DMSO), *N*, *N*-dimethylformamide (DMF) obtained from Shanghai Reagent Chemical Co. were dried over calcium hydride and distilled before use. Uracil (U, 99%), mPEG were purchased from Sigma. Acryloyl chloride (96%, Aladdin), cyclohexanecarbonyl chloride (98%, J&K), potassium tert-butoxide (98%, Aladdin), potassium (99%, Aladdin) and MTX (98%, J&K) were used as received. Glycidol (96%, Aladdin) and triethylamine (AR, Shanghai Reagent Chemical Co.) were purified by distillation before use. 96-Well plates were purchased from Corning Costar. RPMI-1640 Medium was obtained from Sigma–Aldrich. 3-[4,5-Dimethylthiazol-2-yl]-2,5-diphenyltetra-zoliumbromide (MTT), tyrpsin, penicillin-streptomycin and fetal bovine serum (FBS) were obtained from Invitrogen. All other chemicals were of analytical grade and used as received.

### Measurements

^1^H NMR (nuclear magnetic resonance) spectra of all the samples were characterized on a Mercury VX 300 M spectrometer in DMSO-*d_6_* at 25°C using tetramethylsilane (TMS) as an internal reference. Size exclusion chromatography and multi-angle laser light scatting analysis (SEC–MALLS) were used to determine the molecular weight and molecular weight distribution. A dual detector system consisting of a MALLS device (DAWNEOS, Wyatt Technology) and an interferometric refractometer (a differential refractive index detector, Optilab DSP, Wyatt Technology) was used. 0.1 M NaNO_3_ served as the eluent at a ﬂow rate of 0.3 ml/min. The column temperature was fixed at 25°C and the MALLS detector was operated at a laser wavelength of 690 nm.

NP morphology was investigated on the transmission electron microscopy (TEM) machine (JEM-2100 microscope) at an acceleration voltage of 100 keV. The samples were prepared by placing a droplet of micelles solution on a copper grid with formvar film, which was stained by a 0.2% (w/v) phosphotungstic acid solution, and then slowly dried in air before visualization.

The hydrodynamic particle size and size distribution of the micelles were measured by dynamic light scattering (DLS) at 25^o^C using Nano-ZS ZEN3600 (Malvern instruments). Data were shown as mean ± standard deviation based on three independent measurements.

### Synthesis of poly (ethylene glycol)-hyperbranched polyglycerol

**Figure 1.**
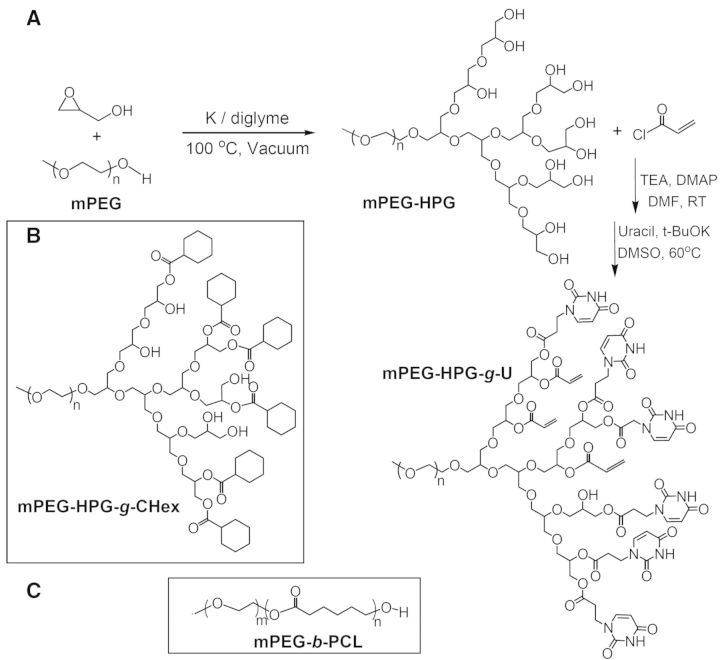
synthetic route of mPEG-HPG-*g*-U (**A**) and chemical structure of mPEG-HPG-*g*-CHex (**B**) and mPEG-*b*-PCL (**C**).

mPEG-HPG was synthesized according to our previously reported method [26]; 4.0 g of mPEG_2000_ (*M*_n_ = 2000 Da, 2.00 mmol) was placed in a triple-neck round bottom flask and dried by heating at 100^o^C for about 5 h under vacuum. After cooling the reaction system, 40 mg of potassium (1.03 mmol) was added and further heated at 100^o^C under reduced pressure for 4 h. At the room temperature, 40 ml of fresh diglyme was added in directly and subsequently 20 ml of glycidol solution in diglyme (0.2 g/ml) was introduced dropwise within 24 h. Upon cooling to room temperature, the reaction was terminated by adding 100 ml of methanol and 8 g of acidic cation exchange resin. The mixture was then filtered and the filtrate was concentrated under reduced pressure. The concentrated solution was poured into a great amount of cool diethyl ether. Collect the precipitate and dry it under vacuum at 50^o^C for 48 h to give a pale yellow solid in 80% yield. ^1^H NMR (DMSO-*d_6_*, 300 MHz), *δ* ppm: *δ* 4.91–4.3 (m, OH, disappeared after D_2_O exchange), 3.84–3.41 (m, CH_2_ and CH), 3.24 (s, OCH_3_) (Fig. 1).

### Synthesis of poly(ethylene-glycol)-hyperbranched polyglycerol-graft-Uralic

mPEG-HPG-*g-*U was synthesized according to the reported methods [27–29]. Brieﬂy, mPEG-HPG (266 mg, 0.1 mmol) and triethylamine (TEA) (0.417 ml, 3 mmol) were dissolved in 5 ml of dry DMF and cooled to 0^o^C. Then, an acryloyl chloride (0.162 ml, 2 mmol) solution in 5 ml of DMF was slowly added within 30 min and the reaction was allowed under stirring overnight. Following the dialysis (RC acetate membrane (MWCO: 1000 Da)) treatment against DMF and deionized water, the solution was freeze-dried to give the product of mPEG-HPG-*g-*Acr in 55% yield. ^1^H NMR (DMSO-*d_6_*, 300 MHz, TMS): *δ* 3.79–3.46, 4.2 (CH_2_ and CH in mPEG-HPG), 3.42 (s, OCH_3_), 5.81–5.93 (t, 1H in vinyl group), 6.05–6.21 (q, 1H in vinyl group), 6.35–6.49 (t, 1H in vinyl group) (Fig. 1).

mPEG-HPG-*g-*Acr (300 mg, 0.1 mmol), U (560 mg, 5 mmol) and potassium tert-butoxide (10 mg) were dissolved in 20 ml of dry DMSO and the solution was allowed for stirring at 60^o^C under N_2_ atmosphere for 3 days [30]. The solution was dialyzed sequentially against DMF and water (MWCO: 1000 Da), and then freeze-dried to provide the product mPEG-HPG-*g-*U (yield 52%). ^1^H NMR (CDCl_3_, 300 MHz, TMS), *δ* ppm: 8.41 (1H, –CON***H***CO–), 7.39 (1H, –NC***H***CH–), 5.65 (1H, –CHC***H***-CO–), 3.88 (2H, –CH_2_C***H***_2_N–), 3.78–3.45, 4.2 (CH_2_ and CH in mPEG-HPG), 3.36 (3H, C***H***_3_–OCH_2_–) (Fig. 1).

### Synthesis of poly (ethylene glycol)-hyperbranched polyglycerol-graft -cyclohexane

mPEG-HPG (266 mg, 0.1 mmol) and TEA (0.278 ml, 2 mmol) were dissolved in 5 ml dry DMF. At 0^o^C, this solution was added slowly with a solution of cyclohexanecarbonyl chloride (0.133 ml, 1 mmol) in 5 ml dry DMF within 30 min and the reaction continued at room temperature overnight. The obtained solution was subjected to dialysis (MWCO: 1000 Da) against a great amount of water. After freeze-drying, mPEG-HPG-*g-*cyclohexane (CHex) was obtained with the yield of 53%. ^1^H NMR (CDCl_3_, 300 MHz): 3.38 (3H, C*H*_3_–OCH_2_–), 3.89–3.45, 4.1–4.2 (CH_2_ and CH in mPEG-HPG), 2.51–2.21 (m, 1H of cyclohexyl group), 2.04–1.15, (5H of cyclohexyl group) (Supplementary Fig. S1).

By comparing the integral area of the signals at *δ* ∼ 3.89–3.45 (CH_2_ and CH in mPEG-HPG) and *δ* ∼ 1.15–2.51 (CH_2_ and CH in cyclohexane), the average number (*x*) of cyclohexyl group per mPEG-HPG molecule was calculated according to the following formula:

a(2000−16)÷44×4+5×(c−1)=b11xx=(1929+5c)b121a,

where *a* is the integral area of the signal at *δ* ∼ 3.89–3.45 ppm (m, CH_2_ and CH in mPEG-HPG), *b* is the integral area of that at *δ* ∼ 1.15–2.51 ppm (CH_2_ and CH in cyclohexane) and *c* is the hydroxyl number of mPEG-HPG.

### Determination of hydroxyl number of mPEG-HPG based on ^1^H NMR spectrometry

The average number of glycidol unit per mPEG-HPG molecule was determined on the basis of ^1^H NMR analysis of the acetate derivative of mPEG-HPG [31]. mPEG-HPG acetate was prepared as following: mPEG-HPG (266 mg, 0.1 mmol) and TEA (0.487 ml, 3.5 mmol) were dissolved in 5 ml of dry DMF. At 0^o^C, a great excess of acetic anhydride (0.189 ml, 2 mmol) in 5 ml of dry DMF was slowly introduced during a period of 30 min. After stirring overnight, the reaction mixture was dialyzed (RC acetate membrane, MWCO: 1000 Da) against water and then freeze-dried to give the product (yield 55%). ^1^H NMR (DMSO-*d_6_*, 300 MHz, TMS): *δ* 3.84–3.45 (m, C*H*_2_ and C*H* adjacent to ether group in mPEG-HPG), 3.44 (s, OC*H*_3_), 1.99 (s, OCOC*H*_3_) (Supplementary Fig. S2).

By comparing the integral area of protons belonging to CH_2_ and CH in mPEG-HPG and that of the terminal methyl group, the number of hydroxyl group and *M*_n_ (number-average molecular weight) of mPEG-HPG could be determined according to the following formula:

a(2000−16)÷44×4+5×(x−1)=b3xx=1929b11×(3a−5b)

$$M_{\text{n}}=\text{74}\times (x-\text{1})+\text{2}000,$$

where *a* is the integral area of the peak at *δ* 3.78–3.45, *b* is the integral area of the peak at *δ* 1.99, *x* is the hydroxyl number of mPEG-HPG and (*x*−1) represents the average number of glycidol unit per mPEG-HPG molecule.

### Synthesis of Uracil-terminated mPEG (mPEG-U)

#### Synthesis of 1-(carboxymethyl) Uracil (U-COOH)

1-(carboxymethyl) Uracil (U-COOH) was synthesized from Uracil according to the literature [32]. Uracil (11.2 g, 0.1 mol), KOH (28 g, 0.5 mol) and bromoacetic acid (27.6 g, 0.2 mol) were dissolved in 125 ml of water and refluxed for 60 min. Cooling the solution to room temperature, the solution pH was adjusted to 2 using 1 M HCl. The resulting precipitate was collected by filtration and in turn washed with water, ethanol and ethyl acetate, respectively. A white powder solid was obtained in 80% yield after drying under vacuum at 30^o^C for 12 h. ^1^H NMR (300 MHz, DMSO-*d_6_*, 25°C, TMS, pm): 4.36 (s, 2H, CH_2)_, 5.7, 7.5 (s, 1H, CH in allyl group), 11.36 (s, 1H, NH) (Supplementary Fig. S3).

#### Synthesis route of mPEG-U

Briefly, mPEG_1000_ (0.25 g, 0.25 mmol), U-COOH (0.213 g, 1.25 mmol) and 4-dimethylaminopyridine (0.024 g, 0.2 mmol) were dissolved in 15 ml of dry DMF and cooled to 0^o^C in ice bath [32]. Then dicyclohexylcarbodiimide (0.36 g, 1.75 mmol) was added slowly within 30 min and the mixture was stirred at room temperature overnight. The reaction solution was then filtered and the solution of the filtrate was removed under reduced pressure. The crude product was dissolved in a small amount of dichloromethane. Remove the insoluble substance by filtration and the filtrate was poured into a great amount of cool diethyl ether. Collect the precipitate and dry it under vacuum at 30^o^C for 24 h to give a white solid in 57% yield. (CDCl_3_, 300 MHz, TMS): *δ* ppm: 8.15 (1H, –CON***H***CO–), 6.47 (1H, –NC***H***CH–), 5.78 (1H, –CHC***H***–CO–), 4.53 (2H, –NC***H***_2_CO), 4.35 (2H, –CH_2_C***H***_2_N–), 3.79–3.41 (m, CH_2_ in mPEG), 3.38 (3H, C***H***_3_–OCH_2_–) (Supplementary Fig. S4).

### Synthesis of mPEG-*block*-poly(caprolactone)

Briefly, 0.1 g of mPEG_2000_, 0.7 g of ε-CL and a predetermined volume 0.1 M Sn(Oct)_2_ solution in toluene ([monomer]:[catalyst] =900 :1) were added in a vessel which was pre-treated with trimethylchlorosilane [31]. The vessel was sealed under vacuum (60 Pa) and immersed into an oil bath thermostated at 100^o^C. After 12 h reaction, the reaction mixture was dissolved in a small amount of chloroform and poured into a large amount of ethyl ether. The precipitates were collected by centrifugation and dried under vacuum (yield 81%). ^1^H NMR (CDCl_3_, 300 MHz, TMS): *δ* 4.14–4.00 (t, C*H*_2_OC=O), 3.75–3.56 (m, 2H, CH_2_ of mPEG), 3.40 (s, OCH_3_), 2.38–2.24 (t, OC=OC*H*_2_CH_2_), 1.75–1.54 (m,OCOCH_2_C*H*_2_CH_2_C*H*_2_ CH_3_), 1.48–1.29 (t, OCOCH_2_CH_2_C*H*_2_CH_2_ CH_3_) (Supplementary Fig. S5).

### Synthesis of 1-octadecylpyrimidine-2,4(1H,3H)-dione (U-C_18_)

Uracil (224.06 mg, 2 mmol) and K_2_CO_3_ (165.6 mg, 1.2 mmol) were added into 10 ml of dry DMF. At 60^o^C, the mixture was added slowly with 10 ml of 1-bromooctadecane (332.21 mg, 1 mmol) solution in DMF within 30 min and then the reaction was allowed for stirring overnight. After filtration, DMF was removed by evaporation under vacuum. The crude product was dissolved in an appropriate amount of dichloromethane. Dichloromethane was removed after the filtration to isolate the insoluble substance. The crude product was recrystallized from ethanol and dried under vacuum for 24 h to give a white solid in 82% yield. ^1^H NMR (CDCl_3_, 300 MHz, TMS), *δ* ppm: 8.64 (1H, –CON*H*CO–), 7.15 (1H, –C*H*CH–CO–), 5.70 (1H, –CHC*H*–CO–), 3.71 (2H, –NC*H*_2_CH_2_), 0.81–1.69 (35H, –C*H*_2_(C*H*_2_)_15_C*H*_3_) (Supplementary Fig. S6).

### mNP fabrication and determination of critical micelle concentration

A predetermined amount of polymers were directly dissolved into 2 ml of distilled water and the solution was kept under stirring overnight. Pyrene was used as a hydrophobic fluorescent probe to measure the critical micelle concentration (CMC) by fluorescence spectroscopy. Briefly, a series of aqueous solutions with different polymer concentration were prepared, in which pyrene concentration was fixed at 6 × 10^−^^7^ M. The solutions were kept at room temperature for 24 h to reach the solubilization equilibrium of pyrene in the aqueous phase. Emission was carried out at 393 nm, and excitation spectra were recorded ranging from 300 to 360 nm. Both emission bandwidth and excitation bandwidth were 5 nm. Fluorescence spectra were recorded on a LS55 luminescence spectrometer (Perkine-Elmer). From the pyrene excitation spectrum, the intensity ratio (*I*_323_/*I*_320_ for mPEG-HPG-*g*-CHex and *I*_338_/*I*_335_ for mPEG-HPG-*g*-U) was plotted against the logarithm of the polymer concentration. The CMC was determined based on the crossover point at low polymer concentration on this plot (Supplementary Fig. S7).

### MTX loading and *in vitro* drug release

A predetermined amount of MTX and polymer samples were dissolved in 5 ml of DMF. The solutions were put into a dialysis tube (RC acetate membrane, molecular weight cut-off: 1000 g/mol) and then dialyzed against 1 l of deionized water for 48 h. During the process, the deionized water was refreshed every 4 h to remove the solvents and the unloaded drugs. After that, the solution was freeze-dried to obtain MTX-loaded micelles. A predetermined of MTX-loaded micelles was dissolved in DMSO, and the drug loading efficiency (DLE) was determined on the basis of the UV absorbance intensity at 290.5 nm, using a standard calibration curve experimentally obtained. Each value was averaged from three independent experiments. The DLE was defined as:

$$\begin{array}{l} \text{DLE}=(\text{weight of drug loaded in micelles}/\\ \text{weight of drug loaded micelles})\times \text{1}00\%. \end{array}$$

Four milliliters of as-prepared MTX-loaded micelle aqueous solution was introduced into a dialysis bag (MWCO: 3500 Da). The dialysis was carried out in 10 ml of phosphate buffer solution (pH = 7.4) or acetate buffer solution (pH = 5.0), respectively. At the predetermined time interval, the external buffer was changed with 10 ml of fresh buffer solution. The amount of MTX released from the micelles was determined on the basis of the UV absorbance at 303 nm, according to the standard calibration curve experimentally obtained. Each value was averaged from three independent experiments.

### Cell culture

KB cells (human mouth epidermal carcinoma cell line) were cultured in RPMI-1640 Media and COS7 cells were cultured in Dulbecco’s modified Eagle’s medium in an atmosphere containing 5% CO_2_ at 37°C. The media contain 10% FBS and 1% antibiotics (50 units/ml streptomycin and 50 units/ml penicillin).

### Cytotoxicity assay

The cytotoxicity of MTX-free and -loaded micelles in KB and COS7 cells was evaluated by MTT assay. For each well in a 96-well plate, the number of KB cells in each well was 6 × 10^3^. After incubation for 24 h (37^o^C, 5% CO_2_), the cells were continuously incubated for 48 h in the culture media containing the polymer with different concentration. After that, the medium was replaced with 200 µl of fresh medium and 20 µl of MTT (3-(4,5-dimethylthiazol-2-yl)-2,5-diphenyl tetrazolium bromide) (5 mg/ml) solution. After incubation for 4 h, the medium was removed and 200 µl of DMSO was added to dissolve the formazan crystals. The optical density (OD) was spectrophotometrically measured in an ELISA plate reader (model 550, Bio-Rad, USA) at a wavelength of 570 nm. We defined the cell viability as:

$$\text{Cell viability}\left(\%\right)=\left({\text{OD}}_{\text{57}0\left(\text{samples}\right)}/{\text{OD}}_{\text{57}0(\text{control)}}\right)\times \text{1}00\%,$$

where OD_570(samples)_ represents the absorbance obtained in the presence of the sample and OD_570(control)_ corresponds to the absorbance in the absence of the sample.

## Result and Discussion

**Figure 2.**
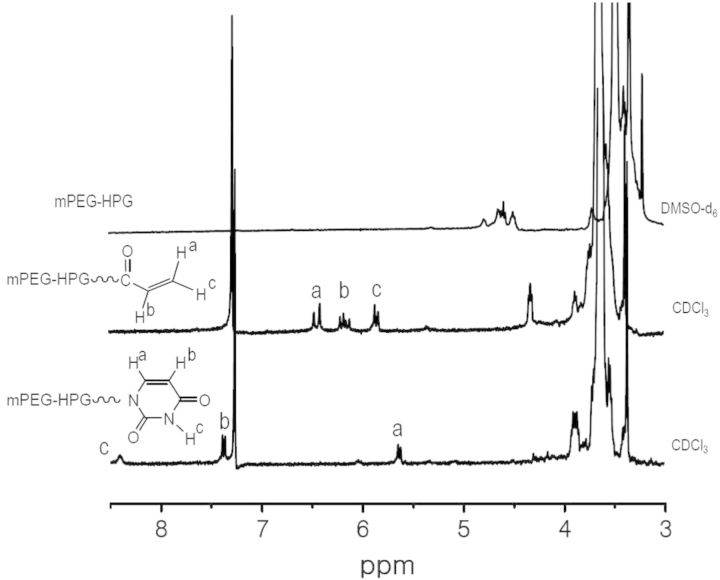
^1^H NMR spectra of mPEG-HPG, mPEG-HPG-*g-*Acr and mPEG-HPG-*g-*U.

A linear-hyperbranched copolymer of mPEG-HPG was firstly prepared through the ring-opening polymerization of glycidol using mPEG (*M*_n_ = 2000 Da) as the macroinitiator [26]. The molecular weight (*M*_n_,_NMR_) of 2660 Da calculated according to proton ^1^H NMR spectrometry agreed well with *M*_n_,_SEC_ (2710 Da) determined by size-exclusion chromatography and multiangle laser light scattering (SEC–MALLS) technique. The terminal hydroxyl groups of mPEG-HPG was converted to vinyl groups by reacting with acryloyl chloride and then subjected to Michael addition reaction with nucleobase Uralic (Fig. 1) [27–29]. The acryloyl intermediate of mPEG-HPG-*g-*Acr and final product of mPEG-HPG-*g*-U were purified by dialysis treatment and the structure was verified by ^1^H NMR spectroanalysis (Fig. 2). By comparing the spectrum of mPEG-HPG-*g*-U with that of mPEG-HPG-*g-*Acr, it was found that the resonances belonging to vinyl groups of the latter almost disappeared and simultaneously there emerged the characteristic signals attributed to U groups. It indicated that U group can be readily conjugated onto polymer chains. Thus, by adjusting the feeding ratio, three mPEG-HPG-*g*-U*_x_* copolymers were eventually fabricated with varied substitution degree of U (*X* = 3.6, 4.6, 6.8). Herein, *X* represented the mean number of Uralic group per mPEG-HPG-*g*-U molecule, which was determined on the basis of UV–vis measurement.

**Figure 3.**
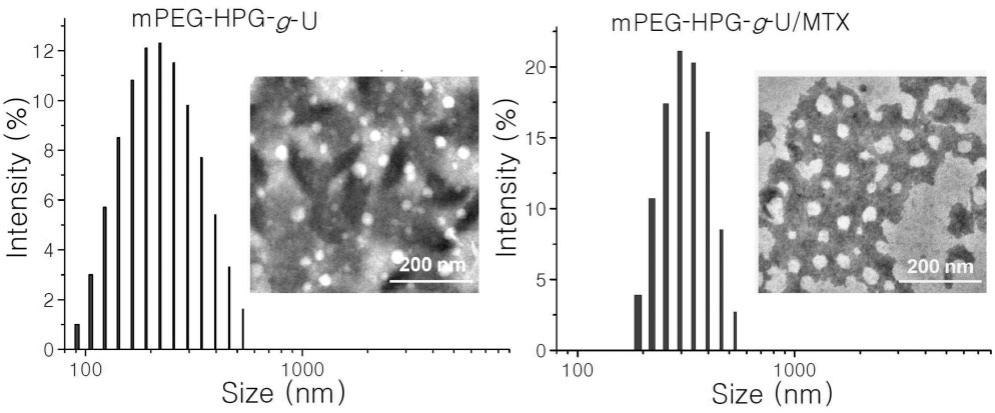
DLS profiles and TEM images of MTX-free (**A**) and MTX-loaded (**B**) mPEG-HPG-g-U NPs.

MTX loading into mPEG-HPG-*g*-U matrix was achieved by the well-established dialysis method. TEM image and DLS profile clearly revealed the nanoscale morphology of the formed mPEG-HPG-*g*-U/MTX complexes (Fig. 3). DLS data indicated that the mean hydrodynamic diameters of the micelles before and after MTX loading were about 200 and 300 nm, respectively, both with a narrow size distribution (PDI < 0.2). TEM images showed that both the MTX-free and -loaded micelles were individually dispersed as spherical NPs in dry state. Relatively, the latter appeared to be larger and more irregular. The significant increase of the particle size suggested that the drug cargo can be effectively loaded into the NP inner.

**Figure 4.**
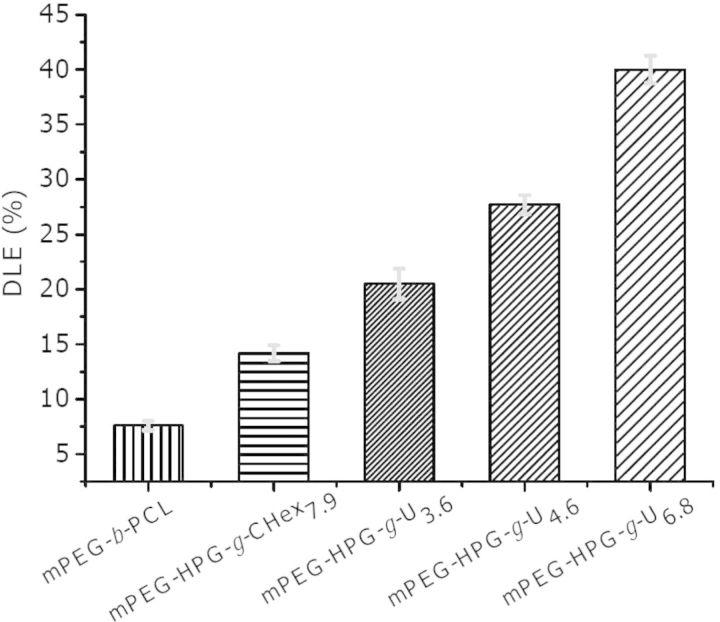
DLE of a series of MTX-loaded NPs.

The DLE of mPEG-HPG-*g*-U was determined on the basis of UV–vis measurement. For comparison, two PEGylated block copolymers were additionally prepared while the chain length of mPEG segment kept unchanged, including linear mPEG-block-polycaprolactone (mPEG-*b*-PCL, *M*_n_,_NMR_ = 3230 and *M*_n_,_SEC_ = 3820 Da) and linear-hyperbranched counterpart of mPEG-HPG-*g*-CHex_7.9_ with the Uralic group being replaced with hydrophobic cyclohexane group (Fig. 1). As shown in Fig. 4, all the linear-hyperbranched copolymers gave the apparently higher DLE values over linear mPEG-*b*-PCL (about 8%), confirming the superiority of hyperbranched architecture in terms of the loading capacity to hydrophobic agents [33]. A gradual increase of DLE was detected as elevating Uralic content in mPEG-HPG-*g*-U*_x_* (DLE ∼ 20.5%, 27.7% and 40.0% for *X* ∼ 3.6, 4.6 and 6.8, respectively). It is noted that despite the similar architecture and substitution degree between mPEG-HPG-*g*-CHex_7.9_ and mPEG-HPG-*g*-U_6.8_, the latter gave the DLE at a much higher level of 40% while the former approximated to 15%. Compared with mPEG-HPG-*g*-CHex_7.9_, mPEG-HPG-*g*-U_3.6_ with a much less substitution degree afforded even better outcome of DLE ∼ 20.5%. To a certain extent, the high DLE of mPEG-HPG-*g*-U_4.6_ NPs agreed fairly well with the finding that a marked increase of 100 nm was detected for the particle size of mPEG-HPG-*g*-U_4.6_ NPs (from 200 to 300 nm) after MTX loading whereas that was just about 50 nm for mPEG-HPG-*g*-CHex_7.9_ NPs (from 150 to 200 nm).

Like typical micelles, hydrophobic interaction ought to be the main driving force responsible for the capability of mPEG-HPG-*g*-CHex_7.9_ NPs to load hydrophobic agents. In general, strong hydrophobicity is favorable for the physical encapsulation of poorly water-soluble agents [8–11]. Relative to mPEG-HPG-*g*-U_6.8_, mPEG-HPG-*g*-CHex_7.9_ possessed stronger hydrophobicity and aggregated into micelles more readily, which was revealed by its considerably lower CMC value and smaller micelle size (95.48 mg/l and 200 nm of mPEG-HPG-*g*-U_6.8_ vs. 21.87 mg/l and 150 nm of mPEG-HPG-*g*-CHex_7.9_). Therefore, there must be other forces in addition to hydrophobic interaction that should account for the unusually higher MTX-loading efficiency of mPEG-HPG-*g*-U. This is thought to be attributed to the ability of MTX to form hydrogen bonds with terminal Uralic groups of mPEG-HPG-*g*-U. Owing to the enhanced hydrophobicity upon MTX conjugation, polymeric matrix would self-aggregate into micelles more readily during the dialysis process. This MTX-conjugated micelle can continuously accommodate MTX cargo via hydrophobic interaction.

**Figure 5.**
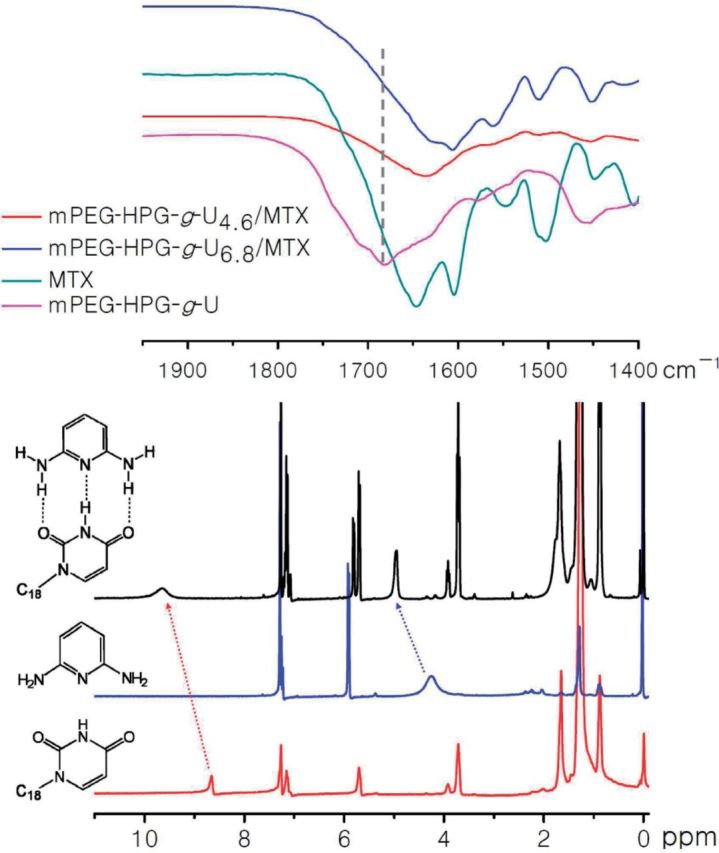
IR spectra of MTX, mPEG-HPG-*g*-U and MTX-loaded NPs (upper) and ^1^H NMR spectra in CDCl_3_ of DPA (red), U-C_18_ (black) and the mixture of DPA and U-C_18_ (below).

IR and ^1^H NMR spectrometry was conducted to demonstrate the H-bond interaction between U and MTX. By comparing IR spectra of mPEG-HPG-*g*-U and MTX-loaded NPs, it was found that the carbonyl stretching peak of –CONH– of mPEG-HPG-*g*-U moved toward the lower frequency after loading MTX into mPEG-HPG-*g*-U matrix (Fig. 5) [11]. Because of the sharply different solubility of MTX and mPEG-HPG-*g*-U in the commonly used deuterated solvents, 1-octadecyl modified Uralic (U-C_18_) and 2,6-aminepyridine (DAP) were used as the molecular models for ^1^H NMR analysis and the spectra were recorded in CDCl_3_. As shown in Fig. 5, there appeared an evident shift of the resonance of N–H proton of U-C_18_ from 8.64 to 9.64 ppm upon the addition of DAP; and that of N–H proton of DAP correspondingly shifted from 4.28 to 4.95 ppm. IR and ^1^H NMR analyses strongly suggested the capability of mPEG-HPG-*g*-U to have H-bond interaction with DAP-bearing MTX.

**Figure 6.**
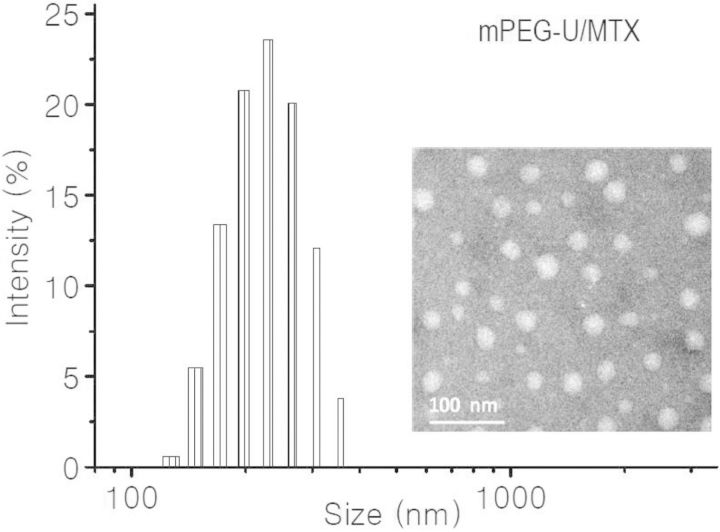
DLS profile (left) and TEM image (right) of mPEG-U/MTX NPs.

To provide more intuitive evidence about the ability of U to form H-bonds with MTX, Uralic-terminated mPEG (*M*_n_ = 1000 Da) (mPEG-U) was dissolved in DMF together with MTX. The organic solution was dialyzed against deionized water to obtain an aqueous solution, in which mPEG-U concentration was fixed at about 0.25 mg/ml. Both the TEM image and DLS profile clearly revealed the production of NPs in the solution (Fig. 6). Noticeably, mPEG-U itself remained absolutely water-soluble at this concentration. It is thus suggested that there must happen the special interaction between mPEG-U and MTX, so the formed complexes can self-assemble into NPs due to the reduced hydrophilicity/hydrophobicity balance ratio after the incorporation of hydrophobic MTX. Taken together, all the experimental results demonstrated that the MTX-loading process involved the mechanism associated with the H-bond interaction between U and MTX.

**Figure 7.**
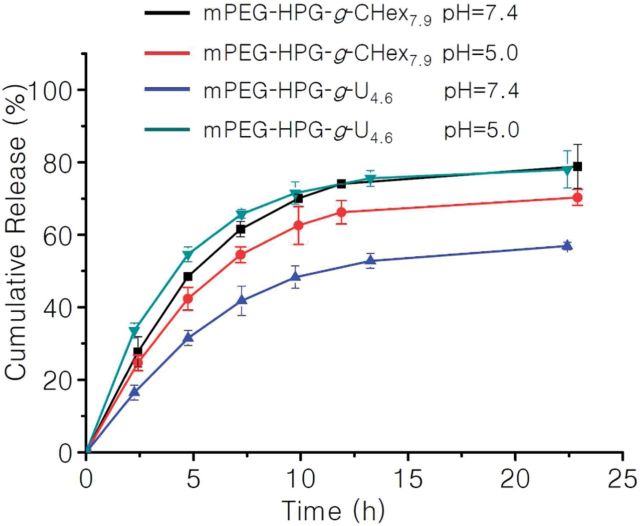
*in vitro* MTX release profiles of MTX-loaded NPs from mPEG-HPG-*g*-U_4.6_ and mPEG-HPG-*g*-CHex_7.9_ in PBS (pH = 7.4) and ABS (pH = 5.0) buffer at 37°C.

After passive accumulation of pH-responsive NPs into tumor tissue followed by the cellular internalization, the acidic microenvironment of late endosome/lysosome organelles (pH = 4–5.5) can be used as a biological stimuli to induce the effective liberation of the loaded drugs inside tumor cells [24, 25]. This is well established to be beneficial for the treatment of tumor chemotherapy. As well documented, acidic environment can affect the protonation of carbonyl groups and amino groups and thus discourage the formation of hydrogen bonds [20–22]. Therefore, it is reasonably expected that mPEG-HPG-*g*-U/MTX delivery system would display a pH-responsive drug release behavior. To elucidate this possibility, the *in vitro* MTX release behavior from mPEG-HPG-*g*-U_4.6_/MTX NPs was investigated in PBS (pH = 7.4) and acetate buffer solution (pH = 5.0) at 37^o^C, respectively (Fig. 7). mPEG-HPG-*g*-U_4.6_ NPs liberated MTX at neutral condition more slowly when compared with the control using mPEG-HPG-*g*-CHex_7.9_ NPs as a carrier. This finding can find the explanation based on the fact that the fraction of conjugated MTX in mPEG-HPG-*g*-U_4.6_/MTX NPs is more difficult to escape away than those physically encapsulated in mPEG-HPG-*g*-CHex_7.9_ NPs. The resultant data in Fig. 7 also indicated that the release of MTX from mPEG-HPG-*g*-U_4.6_ NPs was indeed accelerated in response to the reduced pH. In contrast, pH decline from 7.4 to 5.0 appeared to conversely retard the MTX release from mPEG-HPG-*g*-CHex_7.9_ NPs, possibly due to the larger water-solubility of MTX at a higher pH. In fact, the commercialized MTX injection is always adjusted to basic pH even up to 9.0 so as to achieve a higher drug concentration. At this point, our design seems to possess a superior advantage over the traditional non-functional polymeric mNPs such as mPEG-HPG-*g*-CHex_7.9_.

**Figure 8.**
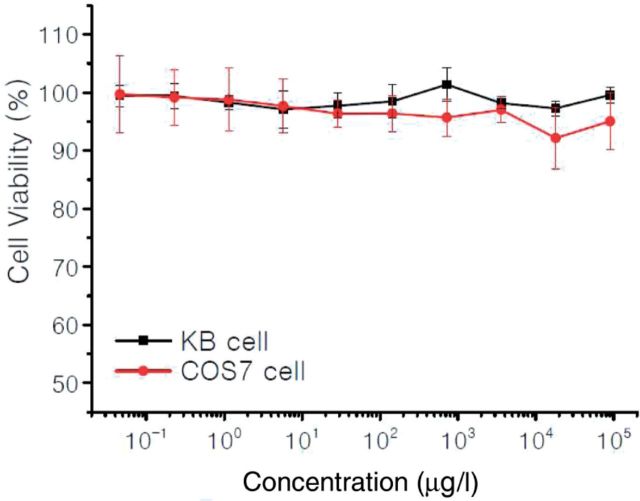
Relative viability of KB and COS7 cells incubated for 48 h with different concentrations of mPEG-HPG-*g*-U_4.6_ NPs.

The acceptable biocompatibility is a necessary requirement for the practical application of biomaterials. The preliminary *in vitro* cytotoxicity of mPEG-HPG-*g*-U_4.6_ was evaluated by MTT assay in KB cells and COS7 cells (Fig. 8). The resultant data indicated that mPEG-HPG-*g*-U_4.6_ have negligible cytotoxicity even at the concentration up to 100 mg/l, suggesting the good cell biocompatibility.

## Conclusion

In summary, the present work described our attempt to enhance the loading efficiency of antitumor MTX in delivery NPs by virtue of special H-bond interaction of Uralic with MTX. Uralic-terminated linear-hyperbranched mPEG-HPG-*g*-U copolymers can form nano-sized micellar complexes with MTX and afforded extremely high loading efficiencies up to 40%. This outcome is considerably better than that of the U-absent mNPs. In addition, mPEG-HPG-*g*-U/MTX NPs were shown to release the entrapped MTX in an acidity-accelerated manner, which would be favorable for the tumor chemotherapy.

## Supplementary Data

Supplementary data is available at *REGBIO Journal* online.
